# Genetic Contribution to the Pathogenesis of Primary Biliary Cholangitis

**DOI:** 10.1155/2017/3073504

**Published:** 2017-02-01

**Authors:** Satoru Joshita, Takeji Umemura, Eiji Tanaka, Masao Ota

**Affiliations:** ^1^Department of Medicine, Division of Gastroenterology and Hepatology, Shinshu University School of Medicine, 3-1-1 Asahi, Matsumoto, Nagano 390-8621, Japan; ^2^Department of Legal Medicine, Shinshu University School of Medicine, 3-1-1 Asahi, Matsumoto, Nagano 390-8621, Japan

## Abstract

Formerly termed primary biliary cirrhosis, primary biliary cholangitis (PBC) is a chronic and progressive cholestatic liver disease characterized by the presence of antimitochondrial antibodies. Ursodeoxycholic acid (UDCA) therapy is the most effective and approved treatment for PBC and leads to a favorable outcome in the vast majority of cases. Although the etiology of PBC has not yet been elucidated, human leukocyte antigen (HLA) class II alleles have been consistently associated with disease onset for decades. Individuals in different geographic regions of the world may have varying susceptibility alleles that reflect indigenous triggering antigens. In this review, we describe the influence of HLA alleles and other gene polymorphisms on PBC along with the results of genome-wide association studies (GWAS) on this disease.

## 1. Introduction 

Primary biliary cholangitis (PBC), formerly known as primary biliary cirrhosis [1, 2], is a liver-specific autoimmune disease characterized by female preponderance and destruction of intrahepatic bile ducts that often results in cirrhosis and hepatic failure [3–5]. The prevalence of PBC ranges from 20 to 40 cases per 100,000 persons [4–6], although the number of patients with PBC, specifically asymptomatic PBC, is on the rise due mainly to increased awareness and earlier detection by disease-specific antimitochondrial antibodies (AMAs). Ursodeoxycholic acid (UDCA) therapy is the most effective treatment for PBC and is recommended by most guidelines [7, 8]. The vast majority of patients with PBC show a favorable response to UDCA treatment despite some cases of disease progression via unknown mechanisms [9, 10]. Genetic factors are considered to play a prominent role in disease onset as higher concordance rates in monozygotic twins than in dizygotic twins and familial clustering of patients with PBC has been demonstrated in family and population studies [11–16]. However, the etiology of this disease has yet to be conclusively clarified; PBC is presumed to be a multifactorial polygenic condition caused by allelic triggers and environmental factors in genetically susceptible individuals, although epigenetic mechanisms, such as instability of X chromosome gene expression, may also participate in the disease's female predominance [17–19].

In the present article, we summarize the literature on human leukocyte antigen (HLA) involvement in PBC onset and GWAS findings from North American, European, and Japanese populations to explore the disease pathways of PBC pathogenesis.

## 2. Associations between HLA and PBC Susceptibility

Many significant susceptibility single nucleotide polymorphisms (SNPs), such as* CTLA4*,* TNF-a*,* STAT4*,* PTPN22*, and* VDR, *have been identified using candidate gene methods [20–24]. Among them, however, only* HLA *has consistently been associated with PBC in distinct patient cohorts across ethnicities.

Located on the most gene-dense genomic region on chromosomal position 6p21 [25], HLA genes are extremely polymorphic and play an essential role in numerous biologically and medically relevant processes. The products of the classical HLA class I (*A, B, *and* C*) and class II (*DR, DQ, *and* DP*) genes include cell-surface glycoproteins involved in the binding and presentation of self- or non-self-peptides to T-cell receptors (TCRs). Class I molecules present endogenous peptides derived from viruses to CD8^+^ cytotoxic T cells, while class II molecules present processed peptides from exogenous pathogens to CD4^+^ helper T cells. The extent of endogenous and exogenous peptide binding to HLA molecules depends on allelic polymorphisms. Additionally, both HLA class I and II molecules have functional roles in protein interactions, transcription regulation involved in the inflammatory response, and natural killer cell-cytokine interactions as part of innate immunity.

HLA polymorphisms have been extensively studied in immune-mediated diseases, revealing associations of particular alleles with ankylosing spondylitis (AS), Behçet's disease (BD), psoriasis, multiple sclerosis (MS), insulin-dependent diabetes mellitus (IDDM), systemic lupus erythematosus (SLE), inflammatory bowel disease (IBD), rheumatoid arthritis (RA), narcolepsy, autoimmune hepatitis (AIH), and autoimmune pancreatitis (AIP) among others. Early investigations on associations between HLA polymorphisms and PBC were carried out more than a quarter-century ago [37]. Based on these findings, subsequent cumulative studies have provided evidence that PBC is associated with* DRB1*^*∗*^*08* as predisposing and* DRB1*^*∗*^*11* and* DRB1*^*∗*^*13* as protective alleles [28, 38]. Li et al. conducted a meta-analysis to assess for relationships between HLA class II and disease susceptibility to PBC and demonstrated that* HLA DR*^*∗*^*07* and ^*∗*^*08* alleles were risk factors for PBC in certain populations, whereas* DR*^*∗*^*11*, ^*∗*^*12*, ^*∗*^*13,* and ^*∗*^*15* alleles were protective factors [39].

Several key reports [26–28] on the association between HLA haplotype and PBC susceptibility or resistance are summarized in Table 1. *HLA DR*^*∗*^*08* alleles caused disease susceptibility, while* HLA DRB1*^*∗*^*13 *and ^*∗*^*11* alleles conferred disease protection in haplotype analyses across ethnicities. Both protective* DRB1*^*∗*^*11* and* DRB1*^*∗*^*13* alleles have also been implicated* DRB1*^*∗*^*11* against hepatitis C [40], human papilloma [41], and human immunodeficiency [42] and* DRB1*^*∗*^*13* against hepatitis C [43], human papilloma [44], and human immunodeficiency [45] viruses along with malaria [44]. Thus, one of the pathogenic mechanisms in PBC may be bacterial infection as these protective HLA class II alleles play a functional role in blocking the invasion of infectious agents. 

However, individuals harboring the above haplotypes constitute only a minority of patients with PBC, suggesting that other candidate genes and environmental cues evoke PBC pathogenesis. Umemura et al. [26] reported the possibility that the distribution of DRB1 amino acid residues encoded by different HLA* DRB1* alleles influenced the binding affinity to antigens, which might also be a predominant factor in PBC susceptibility.

## 3. GWAS on PBC

There have been extensive GWAS in patients with PBC, a number of which documenting significant associations with disease risk. To date, five GWAS [29–33], two Illumina immunochip studies [34, 35], and one genome-wide meta-analysis (GWMA) [36] on PBC have been performed on well-characterized cohorts in North American, European, and Japanese populations (Table 2). These investigations clarified that the HLA class II domain possessed the strongest association with disease susceptibility, particularly at the* HLA-DRB1*,* HLA-DQA1*, and* HLA-DQB1* loci. However, HLA alone does not explain the entire genetic predisposition to PBC, mainly since 80–90% of patients with the disease do not carry the most common HLA susceptibility alleles. In this regard, other genes apart from HLA loci are suggested to contribute to disease development. At present, GWAS have identified 39 non-HLA loci predisposing to PBC at a genome-wide level of significance (Table 3).

The first GWAS [29] in a North American cohort identified a significant association of PBC with genetic variants at* IL12A*, encoding IL-12 p35, and* IL12RB2,* encoding IL-12 receptor *β*2. Modest (*p* < 5.0 × 10^−5^) genome-wide associations with disease risk for SNPs at the* signal transducer and activator of transcription 4 (STAT4)* and* cytotoxic T-lymphocyte-associated protein 4 (CTLA4)* loci were found as well. The second GWAS [31] confirmed the existence of additional risk loci, including* interferon regulatory factor 5 (IRF5)*,* transportin 3 (TNPO3)*, and* SPIB* encoding a transcription factor involved in B-cell receptor signaling and T-cell lineage decisions. A subsequent noteworthy GWAS from Japan showed that the* IL12A* and* IL12RB2* loci were not significantly associated with PBC, but rather that the* TNFSF15* and* POU2AF1* genes constituted novel risk loci in Japanese patients with PBC along with other non-HLA loci, including* IL7R*,* IKZF3*,* CD80*,* STAT4*, and* NFKB1*. This discrepancy among ethnicities indicated important differences in the pathogenesis of PBC despite several common key molecules and pathways, such as the IL-12 pathway to induce Th1 polarization of CD4^+^ T cells. Our body of evidence suggests that there may be an inherited abnormality in immune regulation during PBC onset and perhaps an inability to suppress inflammatory attacks on small bile ducts once initiated.

It should be noted that Juran et al. identified risk-conferring epistatic interactions between* IL12RB2* and* IRF5* loci [34] as well as between* CTLA4* and* TNFα* loci in the pre-GWAS era [46]. Epistatic interactions between genes revealed by GWAS in the pathogenesis of PBC should be explored in future studies.

While gene associations are of considerable interest in the pathogenesis of PBC, virtually none have been translated into useful clinical testing. For instance, the importance of the IL-12 pathway in PBC onset has been highlighted in animal models and in the case of a child with a congenital IL-12 deficiency who developed PBC [47]. Although antibodies or drugs targeting the IL-12 pathway would seem to be effective, clinical trials using ustekinumab, a human monoclonal antibody directed against IL-12 and IL-23, have failed to produce effects in phase II trials [48]. One reason explaining the discrepancy between GWAS results and clinical testing may be that clinicians typically encounter patients who have already become complicated with cholestasis; in fact, the immunological destruction of cholangiocytes occurs in the very early stages of PBC. Thus, the mechanisms of disease progression should also be addressed to halt the deterioration of disease status and afford PBC patients an improved prognosis.

Lastly, it is particularly interesting that many genes implicated in PBC pathogenesis by GWAS have also been reported in other autoimmune diseases, such as SLE, systemic sclerosis (SSc), and Sjögren's syndrome (Table 3), suggesting a genetic overlap. Understanding the mechanisms involved in the onset and progression of certain autoimmune diseases may accordingly shed light on those in PBC.

## 4. Conclusions and Future Directions

The pathogenesis of PBC is incompletely understood but appears to involve genetic susceptibility and resistance alleles in HLA and other gene loci, with a possible overlap with several autoimmune diseases. It is also probable that genetically susceptible individuals develop PBC following environmental cues, leading to both adaptive and innate immune responses that result in portal inflammation and bile duct epithelial damage. In addition to susceptibility, the precise mechanisms of PBC progression should be addressed to improve patient prognosis and quality of life.

## Figures and Tables

**Table 1:** HLA haplotype associations with PBC.

Study	Population	HLA allele	*p* value	OR (95% CI)
Susceptibility				

Umemura et al. [26]	Japanese	DRB1^*∗*^08:03-DQB1^*∗*^06:01	0.000025	2.22 (1.53–3.20)
		DRB1^*∗*^04:05-DQB1^*∗*^04:01	0.044	1.38 (10.2–1.87)
Zhao et al. [27]	Chinese	DRB1^*∗*^08:03-DQB1^*∗*^06:01	<0.0001	3.17 (1.91–5.23)
		DRB1^*∗*^07:01-DQB1^*∗*^02:02	0.005	1.85 (1.20–2.83)
Donaldson et al. [28]	UK	DRB1^*∗*^08:01-DQA1^*∗*^04^*∗*^01-DQB1^*∗*^04:02	0.0027	2.9
	Italian	DRB1^*∗*^08:01-DQA1^*∗*^04^*∗*^01-DQB1^*∗*^04:02	0.0086	3.41

Protective				

Umemura et al. [26]	Japanese	DRB1^*∗*^13:02-DQB1^*∗*^06:04	0.00093	0.27 (0.12–0.60)
		DRB1^*∗*^11:01-DQB1^*∗*^03:01	0.03	0.37 (0.15–0.88)
Zhao et al. [27]	Chinese	DRB1^*∗*^12:02-DQB1^*∗*^03:01	0.015	0.43 (0.22–0.86)
Donaldson et al. [28]	UK	DRB1^*∗*^11:01-DQA1^*∗*^05:01-DQB1^*∗*^03:01	0.086	0.47
	Italian	DRB1^*∗*^13:01-DQA1^*∗*^01:03-DQB1^*∗*^06:03	0.0041	0.28

**Table 2:** GWAS on PBC.

Study	Year	Platform	Patients	Controls
Hirschfield et al. [29]	2009	Illumina HumanHap 370	1,031	2,713
Hirschfield et al. [30]	2010	Illumina HumanHap 370	1,351	4,700
Liu et al. [31]	2010	Illumina 610K	945	4,651
Mells et al. [32]	2011	Illumina 660W-Quad	1,840	5,163
Nakamura et al. [33]	2012	Affymetrix Axiom	1,274	1,091
Juran et al. [34]	2012	Immunochip	2,426	5,731
Liu et al. [35]	2012	Immunochip	2,861	8,514
Cordell et al. [36]	2015	GWMA	2,764	10,475

**Table 3:** Non-HLA risk loci identified through GWAS as associated with PBC at the genome-wide level of significance.

Chromosome	Locus	Study [reference #]	SNP	OR	*p* value	Candidate gene(s)	Disease(s) with shared risk loci
1	1p31	[35]	rs72678531	1.61	2.47*E* − 38	*IL12RB2*	BD
	1p36	[30]	rs3748816	1.33	3.15*E* − 08	*MMEL1*	MS
	1q31	[32]	rs12134279	1.34	2.06*E* − 14	*DENND1B*	CD

2	2q12	[36]	rs12712133	1.14	5.19*E* − 09	*IL1RL1, IL1RL2, *	
	2q12	[34]	rs10186746	1.21	2.40*E* − 05	*IL1RL1, IL1RL2, *	
	2q32	[32]	rs10931468	1.50	2.35*E* − 19	*STAT4, STAT1*	RA, SLE, Sjögren's, IBD, SSc, BD
	2q36	[36]	rs4973341	1.22	2.34*E* − 10	*CCL20*	

3	3p24	[32]	rs1372072	1.20	2.28*E* − 08	*PLCL2*	RA
	3q13	[35]	rs2293370	1.39	6.84*E* − 16	*CD80*	MS, SLE, Celiac
	3q25	[35]	rs2366643	1.35	3.92*E* − 22	*IL12A*	Celiac

4	4p16	[36]	rs11724804	1.22	9.01*E* − 12	*DGKQ*	
	4q24	[32]	rs7665090	1.26	8.48*E* − 14	*NFKB1*	UC

5	5p13	[35]	rs6871748	1.30	2.26*E* − 13	*IL7R*	MS, UC
	5q21	[36]	rs526231	1.15	1.14*E* − 08	*C5orf30*	
	5q33	[36]	rs2546890	1.15	1.06*E* − 10	*IL12B, LOC285626*	

6	6q23	[36]	rs6933404	1.18	1.27*E* − 10	*OLIG3, TNFAIP3*	
	6q23	[34]	rs6920220	1.29	1.17*E* − 06	*OLIG3, TNFAIP3*	

7	7p14	[32]	rs6974491	1.25	4.44*E* − 08	*ELMO1*	RA, Celiac
	7q32	[35]	rs35188261	1.52	6.52*E* − 22	*IRF5*	RA, SLE, SSc, UC

8	8q24	[34]	rs2608029	1.23	3.14*E* − 06	*PVT1, GSDMC*	

9	9p32	[33]	rs4979462	1.57	1.85*E* − 14	*TNFSF15*	UC, CD

11	11q13	[32]	rs538147	1.23	2.06*E* − 10	*RPS6KA4*	IBD
	11q13	[34]	rs10898201	1.31	4.91*E* − 06	*NADSYN1*	
	11q23	[33]	rs4938534	1.38	3.27*E* − 08	*POU2AF1*	
	11q23	[35]	rs80065107	1.39	7.20*E* − 16	*CXCR5, DDX6*	RA, IBD, Celiac

12	12p13	[35]	rs1800693	1.27	1.18*E* − 14	*TNFRSF1A, LTBR*	MS
	12q24	[35]	rs11065979	1.20	2.87*E* − 09	*SH2B3*	RA, T1DM, Hyperthyroidism, Celiac
	12q24	[34]	rs7309325	1.26	2.54*E* − 05	*SH2B3*	RA, T1DM, Hyperthyroidism, Celiac

13	13q14	[34, 35]	rs3862738	1.33	2.18*E* − 08	*TNFSF11*	CD

14	14q24	[35]	rs911263	1.26	9.95*E* − 11	*RAD51B*	RA
	14q32	[32]	rs8017161	1.22	2.61*E* − 13	*TNFAIP2*	

16	16p13	[35]	rs12708715	1.29	2.19*E* − 13	*CLEC16A, SOCS1*	MS, UC, T1DM
	16q24	[32]	rs11117432	1.31	4.66*E* − 11	*IRF8*	MS, IBD, RA, SSc

17	17q12	[35]	rs17564829	1.26	6.05*E* − 14	*IKZF3*	UC, CD, RA, T1DM
	17q21	[35]	rs17564829	1.25	2.15*E* − 09	*MAPT*	

19	19p12	[35]	rs34536443	1.91	1.23*E* − 12	*TYK2*	IBD, RA, SLE, psoriasis, T1DM
	19p13	[34]	rs73003205	1.35	1.43*E* − 05	*KIAA1683*	
	19q13	[31]	rs3745516	1.46	7.97*E* − 11	*SPIB*	

22	22q13	[35]	rs2267407	1.29	1.29*E* − 13	*SYNGR1*	

CD, Crohn's disease; UC, ulcerative colitis; T1DM, type 1 diabetes mellitus.
